# Does Birth Weight Influence Physical Activity in Youth? A Combined Analysis of Four Studies Using Objectively Measured Physical Activity

**DOI:** 10.1371/journal.pone.0016125

**Published:** 2011-01-12

**Authors:** Charlotte L. Ridgway, Søren Brage, Stephen J. Sharp, Kirsten Corder, Kate L. Westgate, Esther M. van Sluijs, Ian M. Goodyer, Pedro C. Hallal, Sigmund A. Anderssen, Luis B. Sardinha, Lars Bo Andersen, Ulf Ekelund

**Affiliations:** 1 MRC Epidemiology Unit, Institute of Metabolic Science, Cambridge, United Kingdom; 2 Developmental Psychiatry, University of Cambridge, Cambridge, United Kingdom; 3 Federal University of Pelotas, Pelotas, Brazil; 4 Department of Sports Medicine, Norwegian School of Sports Sciences, Oslo, Norway; 5 Faculty of Human Movement, Technical University of Lisbon, Lisbon, Portugal; 6 Institute of Sports Science & Clinical Biomechanics, University of Southern Denmark, Odense, Denmark; Penang Medical College, Malaysia

## Abstract

Animal models suggest growth restriction in utero leads to lower levels of motor activity. Furthermore, individuals with very low birth weight report lower levels of physical activity as adults. The aim of this study was to examine whether birth weight acts as a biological determinant of physical activity and sedentary time. This study uses combined analysis of three European cohorts and one from South America (n = 4,170). Birth weight was measured or parentally reported. Height and weight were measured and used to calculate Body Mass Index (BMI). PA was objectively measured using accelerometry for ≥3 days, ≥10 hours day. Data was standardized to allow comparisons between different monitors. Total physical activity was assessed as counts per minute (cpm), with time spent above moderate activity (MVPA) >2,000 counts and time spent sedentary (<100 counts). There was no evidence for an association between birth weight and total physical activity (p = 0.9) or MVPA (p = 0.7). Overall there was no evidence for an association between birth weight and sedentary time (p = 0.8). However in the Pelotas study we did find an association between higher birth weight (kg) and lower overall physical activity (cpm) (β = −31, 95%CI: −58, −46, p = 0.03) and higher birth weight and greater sedentary time (mins/day) (β = 16.4, 95%CI: 5.3, 27.5, p = 0.004), although this was attenuated and no longer significant with further adjustment for gestational age. Overall this combined analysis suggests that birth weight may not be an important biological determinant of habitual physical activity or sedentary behaviour in children and adolescents.

## Introduction

Lower birth weight individuals may be at greater risk of metabolic diseases in later life, such as diabetes [1] and cardiovascular disease [2] and there is evidence that lower birth weight is associated with higher cardiovascular risk factors in youth [3]. Increasing physical activity may be of particular benefit to those with low birth weight by reducing metabolic risk [4], [5], [6], [7]. However lower birth weight has been associated with reduced physical performance, including muscle strength [8], [9], [10], muscle endurance [11] and aerobic fitness in both childhood [12]–[13] and adulthood [11] and it has been suggested that this lower physical capacity may lead to reduced levels of physical activity [14].

There is some evidence from animal models suggesting that growth restriction in utero can lead to reduced motor activity [15], [16]. There also is evidence that individuals born with extremely low birth weight (<800 g) report less participation in sports activities and lower levels of physical activity compared to normal weight peers during adolescence, despite reporting similar levels of enjoyment of sports [17]. Previous studies in adults, also using self reported physical activity levels, have noted that leisure time activity was lower in those with very low birth weight (<1.5 kg) [18], [19] and birth weight was negatively correlated with reported exercise intensity and frequency of leisure time activity [20]. A recent meta-analysis of the association between birth weight and self reported leisure time physical activity in adolescents and adults suggested an inverted u-shaped relationship, with lower reported activity levels both in those born with low birth weight and in those born at the higher extremes of the birth weight range [14]. Only one previous study in children has used objective monitoring of physical activity and did not detect any associations between birth weight and levels of physical activity [21].

The aim of this study was to examine whether birth weight acts as a biological determinant of physical activity levels across normal birth weight ranges, using a combined analysis of data from four cohort studies with objectively measured physical activity. We investigated the associations between birth weight and physical activity, not only in terms of overall physical activity, but also sub-components (i.e. time spent at moderate and vigorous intensity activity) of physical activity, as well as the association between birth weight and objectively measured sedentary time.

## Methods

### Ethics Statement

All studies were approved by their respective local medical ethics committees and all participants provided informed parental consent and where appropriate informed child assent.

### Description of cohorts

The ‘European Youth Heart Study’ (EYHS) is a large population based cohort from four European countries; Denmark, Norway, Portugal and Estonia, aimed at investigating lifestyle, environmental, and socio-cultural factors associated with cardio-metabolic risk. The sampling procedure, methods and measurements have previously been described in detail [22]. EYHS consists of two age groups, 9 years and 15 years. The EYHS population included 2,928 individuals with retrospective maternally reported birth weight data, of which134 were excluded due to a birth weight <1.5 kg (to exclude those born with very low birth weight, as these are most likely to have existing health issues and may have been born very prematurely, as information on gestational age was not available). Complete birth weight and valid physical activity data were available for a total of 1,240 individuals.

The ‘Roots Study’ is a cohort of adolescents, aged 13–15 years at the time of physical activity measurement, selected from schools within the Cambridgeshire region of the UK; the cohort profile has been previously described in detail [23]. Birth weight was retrospectively maternally reported. We excluded 70 individuals with birth weight <1.5 kg, leaving 811 individuals with valid physical activity data.

The ‘Speedy Study’ is a cohort of younger children aged 9–10 years from Norfolk in the UK, which has been previously described [24]. We included all children with retrospective maternally reported birth weight >1.5 kg (115 were excluded with birth weight <1.5 kg) and valid objectively measured physical activity (n = 1,647).

The ‘Pelotas 1993 Birth Cohort’ is a population based birth cohort in Southern Brazil [25]. In the Pelotas 1993 birth cohort (n = 5,249) birth weight was measured in hospital. We included a sub-sample from the Pelotas 1993 birth cohort in which objectively measured physical activity at age 12–14 years was available. Of these 48 individuals were excluded with birth weight <1.5 kg (n = 472).

### Physical Activity

#### Data collection

Physical activity data was collected using accelerometry in all four studies. EYHS used a 7164 Actigraph monitor, whereas Pelotas and Speedy used a later version of this monitor, the GT1M Actigraph (MTI Actigraph, Manufacturing Technology, Fort Walton Beach, FL, USA). The Actigraph monitors, used in EYHS, Speedy and Pelotas were worn at the hip using an elastic waist band. The Roots study used a combined heart rate and uniaxial accelerometer (Actiheart, CamNtech Ltd, Cambridge) mounted on the chest using ECG electrodes to allow collection of heart rate data in addition to a vertical accelerometer. See table 1 for monitor details.

**Table 1:** Monitor types and protocol.

Study	Monitor	Epoch	Wear protocol	Excluded Zeros	Valid day	No. Days	Cut-points for sedentary	Cut-Points for MVPA	Total activity (counts per minute)
EYHS	7164	1 min	Day time	>10 mins	≥600 mins	≥3 days	<100	>2,000	7164 cpm
Roots	Actiheart	1 min	24 hours	>60 mins	≥600 mins	≥3 days	<20*	>400*	Actiheart cpm*5
Speedy	GT1M	1 min	Day time	>10 mins	≥500 mins	≥3 days	<100	>2,000	GT1M cpm/0.91
Pelotas	GT1M	1 min	24 hours	>60 mins	≥600 mins	≥3 days	<100	>2,000	GT1M cpm/0.91

* A conversion factor of 5.0 was applied to the accelerometry cut points for the Actiheart to make it comparable to 7164 Actigraph monitor cut points.

#### Data conversion

Output from different physical activity monitors is not directly comparable [26]. A previous validation study in adolescents, suggests that the two models of Actigraph used in this analysis, the GT1M and 7164, are largely comparable for time spent at different intensity thresholds, but that when comparing total activity levels (as counts per minute) a conversion factor of 0.91 should be applied (Actigraph 7164 cpm  =  Actigraph GT1M cpm/0.91) [27]. This conversion factor was therefore applied to the studies using the GT1M monitor for total activity (cpm) (Pelotas and Speedy).

As the physical activity data collected the Roots study differed, not only in terms of the monitor itself but also the monitor placement, we developed a suitable conversion factor to apply to the acceleration data from the combined movement and heart rate sensor to ensure it was comparable to the two Actigraph monitors used within the other three studies. Comparable cut points for time spent sedentary, and in moderate activity and vigorous intensity activity were estimated using a laboratory protocol where the volunteers simultaneously wore a 7164 accelerometer and a combined movement and heart rate sensor while walking and running on a treadmill [28]. The laboratory study suggested a conversion factor of 5 (Actigraph 7164 counts  =  Actiheart counts x 5). In order to investigate further whether this conversion was appropriate for free living data we used an existing data set of children, aged 12 to 13 years (mean age 13.1 years) and adolescents aged 16 to 17 years (mean age 17.1 years) who concurrently wore both an combined movement and heart rate sensor and a 7164 Actigraph during free living (n = 50). We compared the total cpm from both Actigraph 7164 and the Actiheart accelerometry, for each participant for each full day of monitor wear (n = 254 days). The data had a skewed distribution, because of the higher ranges at greater intensities, but the median conversion across all the days was 5.16, which was close to the laboratory study conversion factor of 5. Due to the comparability with the laboratory derived estimate, a conversion factor of 5 was used for both total activity (cpm) and time at different intensity thresholds.

#### Data processing

Raw acceleration data files from each individual were processed using a bespoke computer programme (MAHUffe, http://www.mrc-epid.cam.ac.uk/Research/Programmes/Programme_5/InDepth/Programme%205_Downloads.html). All data was processed in 60 second epochs. Missing data or monitor ‘non worn time' was assumed from continuous runs of zero activity counts. For the EYHS and Speedy studies, we excluded runs of zeros >10 mins using the MAHUffe program. Two studies (EYHS and Speedy) asked volunteers to wear their physical activity monitor during waking hours only, while the other two studies (Roots and Pelotas) had 24 hour wear protocols. To avoid the data being unduly influenced by the increased wear time, accelerometry data was excluded for the overnight period between 23:00 hours and 07:00 hours for the two studies that employed a 24 hour protocol (Roots and Pelotas). For Pelotas and Roots the MAHUffe programme was used to generate hourly data and thereafter manually processed in a statistical programme to exclude all data between 23:00 and 7:00. Therefore, any runs of >60 mins of zeros were excluded as ‘non worn time’ before further analyses.

All days with >10 hours were considered valid and participants were included if they had ≥3 valid days of data.

### Summary variables

The following summary outcomes variables were derived from accelerometry data: Total overall physical activity, calculated as total accerometery counts over the wear period (counts per min ‘cpm’). Since cpm is dependant on the wear time a ‘valid day’ was restricted to those recording >10 hours of accelerometry data.

Time spent in moderate and vigorous activity (MVPA) was calculated as time spent above 2,000 counts per minute [29] and sedentary time was calculated as time spent below 100 counts per minute [30] for the 7164 and GT1M Actigraphs.

### Confounding variables

All studies provided a self-reported parental measure of socio-economic status (SES). EYHS combined the mean of parental income and parental education level, categorised from 3–16. For the Roots study 5 categories based on parental wealth and employment were used, while the Speedy study categorised parental education into 6 groups from no qualifications through to degree/post graduate. Finally Pelotas, used years of maternal education, categorised into three groups 0–4, 5–8, ≥9.

All studies also measured both height and weight according to standard anthropometric protocols, described in detail elsewhere: EYHS [22], Roots [23], Speedy [24] and Pelotas [25]. Height and weight data were used to derive Body Mass Index (BMI = weight/height^2^).

### Statistical analyses

Mean and standard deviations are shown for the key descriptive variables, including testing for differences between the four study populations, using one-way ANOVA. To allow comparisons across the differing age ranges of the four study populations, age and sex specific z-scores were created using the WHO Child Growth Standards 2007 [31].

The associations between birth weight, modelled as a continuous variable, and components of physical activity (cpm, time spent in MVPA) and sedentary time were estimated by multiple linear regression analysis separately within each study. Initial models included adjustment for age, sex and monitor worn time. Models were then repeated additionally adjusting for SES and then with further adjustment for BMI to investigate whether overall adiposity influenced any observed associations. To investigate any gender interactions, an interaction term of ‘birth weight x sex’ was added to the model. No significant interaction was observed for any of the studies (p>0.05). Therefore all analysis was performed in the whole dataset, adjusting for sex.

Associations between birth weight (quartiles) and components of objectively measured physical activity within each individual study are displayed graphically. These were presented as figures, with means and 95% CI for each quartile, adjusted for sex, age, SES, monitor wear time and BMI.

The beta coefficients were then combined across studies using random effects meta-analysis, and forest plots were used to display the study-specific and combined estimates of association and 95% confidence intervals. Analyses were performed using SPSS v14.0 (SPSS, Illinois, USA) and Stata version 10 (StataCorp LP, Texus, USA).

## Results

Descriptive statistics (mean and SD) for each of the four studies are displayed in Table 2, all variables, except birth weight, were significantly different between the four study populations. Mean birth weight and SD birth weight were largely comparable across all four studies, with Pelotas having the lowest mean birth weight. Mean age ranged from 10.2 years in the Speedy study up to 14.5 years in the Roots study. Age and sex specific z-scores, using WHO Child Growth Standards, illustrate that all four studies have a mean z-score slightly above the standard, with Speedy and Pelotas having the highest BMI z-scores. Total activity (cpm) was highest in the Speedy study and children in the Speedy study also spent more time in MVPA. Sedentary time was highest in the Roots and Pelotas cohorts, however this is partly explained by greater duration of monitor wear due the 24 hour wear protocol in these studies.

**Table 2:** Descriptive statistics for all four studies included in the combined analysis.

	EYHS		Roots		Speedy		Pelotas		ANOVA
	mean	(SD)	mean	(SD)	mean	(SD)	mean	(SD)	p
Birth weight (kg)	3.46	(0.55)	3.41	(0.52)	3.37	(0.54)	3.22	(0.53)	0.16
Age (years)	12.0	(2.9)	14.5	(0.5)	10.2	(0.3)	13.3	(0.3)	<0.001
Height (m)	1.50	(0.16)	1.67	(0.08)	1.41	(0.07)	1.58	(0.08)	<0.001
Weight (kg)	43.1	(14.8)	57.70	(10.72)	36.59	(8.35)	51.1	(11.9)	<0.001
Body Mass Index (kg/m^2^)	18.6	(3.2)	20.6	(3.4)	18.2	(3.1)	20.3	(3.8)	<0.001
BMI z-score*	0.16	(1.04)	0.19	(1.04)	0.47	(1.15)	0.34	(1.17)	<0.001
Total physical activity (cpm)	630	(234)	406	(150)	735	(243)	487	(167)	<0.001
MVPA (mins/day)	69.2	(40.5)	50.3	(27.2)	73.5	(24.6)	53.0	(31.3)	<0.001
Sedentary time (mins/day)	336.1	(92.3)	550.9	(87.9)	457.3	(54.0)	566.8	(88.7)	<0.001
Number of participants (Boys %)	1,240	(47.4%)	811	(44%)	1,647	(43.9%)	472	(52.4%)	

* Body Mass Index z-score based on age and sex adjusted data using the WHO Child Growth Standards 2007.

### Total Physical Activity

**Figure 1:**
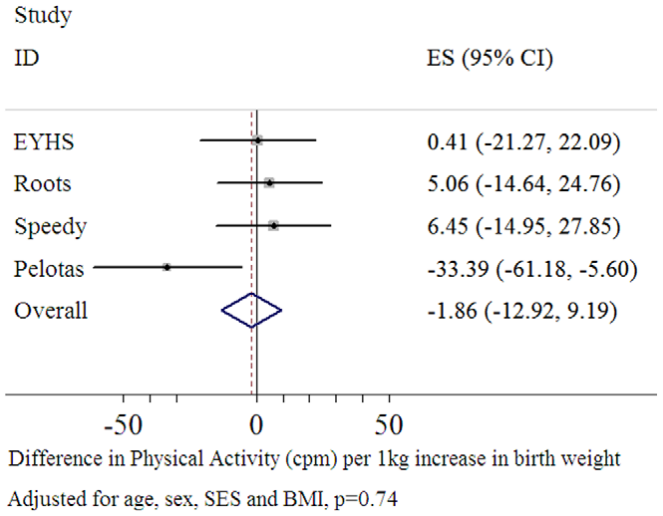
Forest Plot of the association between birth weight and total physical activity (cpm) (n = 4,170).

Results from the combined analysis suggested that there was no significant association between birth weight and objectively measured total physical activity (cpm), with a positive point estimate, but overlapping the null (p = 0.9) (Figure 1), when adjusted for age, sex, monitor worn time, BMI and SES.

**Figure 2:**
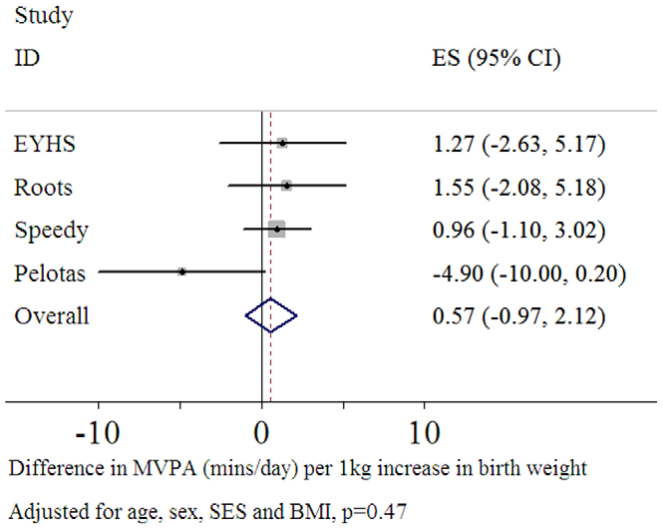
Association between quintiles of birth weight and total physical activity (cpm) in the Pelotas Birth Cohort (n = 472). Means and 95% confidence intervals are adjusted for age, sex, SES, monitor worn time and BMI. (p for trend = 0.0.03).

Although the 95% confidence intervals for three of the studies overlapped the null, the Pelotas study did show a significant association between higher birth weight and lower levels of total physical activity. In this study a 1 kg increase in birth weight was associated with 34 less cpm (β = −31%CI: −61, −6, p = 0.01) adjusted for age, sex and monitor worn time (Table 3). After additional adjustment for SES and BMI the magnitude of association was slightly attenuated but remained statistically significant (β = −31, 95%CI: −58, −4, p = 0.03). When this association was modelled graphically, using quintiles of birth weight, the association appeared to be largely driven by those at the lower end of the birth weight spectrum (Figure 2). We thereafter adjusted our model for gestational age, and the association between birth weight and total physical activity (cpm) was attenuated (β = −29, 95%CI: −60, 2, p = 0.07) (Table 3).

**Table 3:** Regression models for the associations between birth weight and physical activity outcomes within the Pelotas Birth Cohort (n = 472).

	β	95% CI	P
**Total Activity (cpm)**			
Model 1	−33.7	−61.1, −6.3	0.016
Model 2	−32.6	−59.7, −5.6	0.018
Model 3	−30.9	−58.2, −3.8	0.027
Model 4	−29.4	−60.3, 2.2	0.07
**MVPA (mins/day)**			
Model 1	−4.6	−9.6, 0.3	0.07
Model 2	−4.5	−9.5, 0.5	0.08
Model 3	−3.9	−9.0, 1.1	0.1
Model 4	−5.0	−10.7, 0.8	0.09
**Sedentary (mins/day)**			
Model 1	17.0	5.8, 28.2	0.003
Model 2	16.4	5.3, 27.4	0.004
Model 3	16.3	5.2, 27.5	0.004
Model 4	12.2	−0.5, 25.0	0.059

Model 1 – Age, sex, monitor worn time

Model 2 – Age, sex, monitor worn time, plus SES

Model 3 – Age, sex, monitor worn time, SES plus BMI

Model 4 – Age, sex, monitor worn time, SES, BMI, plus gestational age

β represents difference in physical activity outcome per 1 kg increase in birth weight

### Time Spent in Moderate and Vigorous Activity

**Figure 3:**
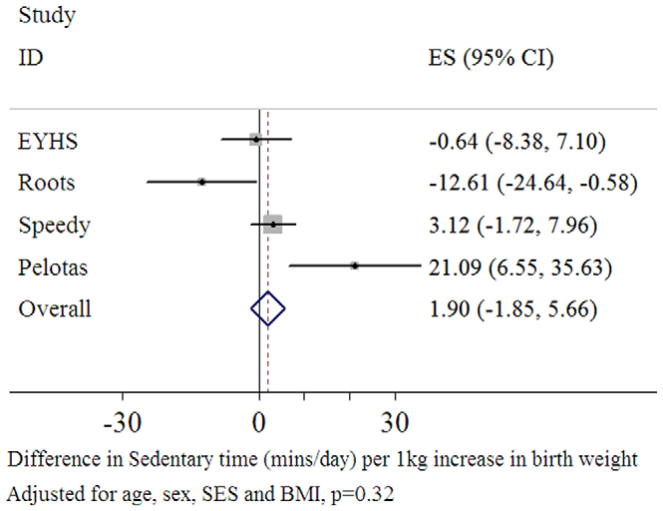
Forest plot of the association between birth weight and moderate and vigorous physical activity (mins/day) (n = 4,170).

There were no significant associations between birth weight and time spent in MVPA (Figure 3), with the estimate from the combined analysis being positive but crossing the null (p = 0.7) (Figure 2), adjusted for age, sex, SES, monitor worn time and BMI. The 95% confidence interval overlapped the null for all four studies. There was also no evidence for any gender interaction within any of the individual studies (p>0.3).

### Time Spent in Sedentary Activity

**Figure 4:**
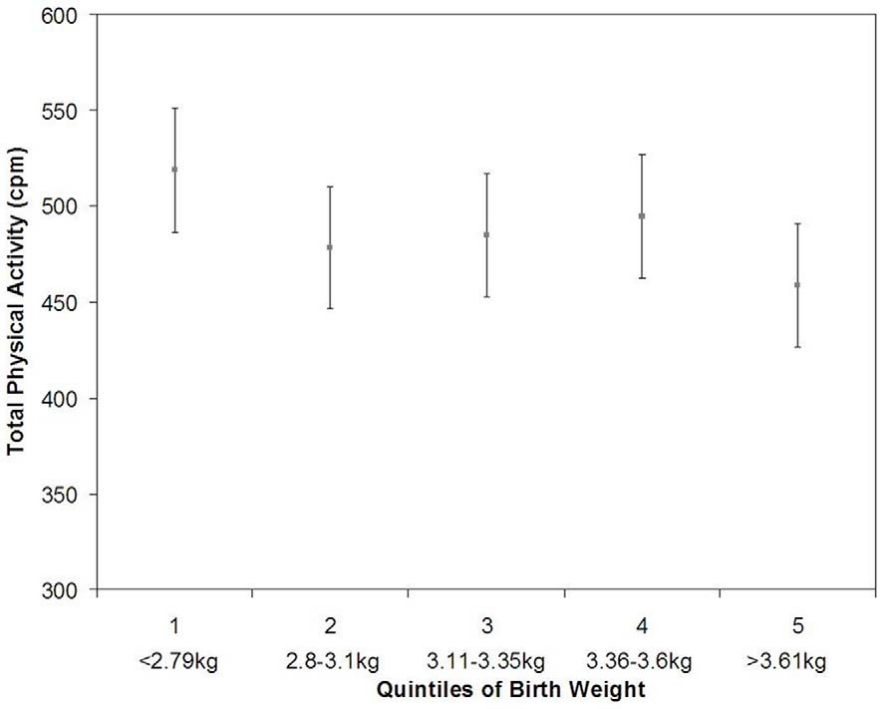
Forest plot of the association between birth weight and sedentary time (mins/day) (n = 4,170).

There was no overall association between birth weight and sedentary time (p = 0.8) Figure 4), adjusted for age, sex, SES, monitor worn time and BMI. There was also no evidence for any gender interaction within any of the four studies (p>0.7).

**Figure 5:**
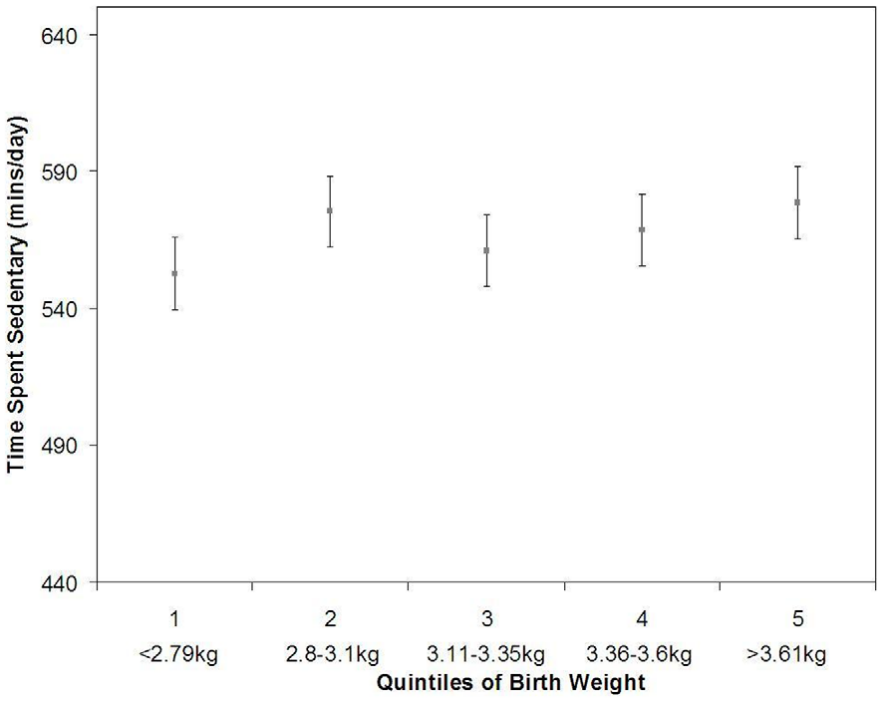
Association between quintiles of birth weight and sedentary time (mins/day) in the Roots Study (n = 747). Means and 95% confidence intervals are adjusted for age, sex, SES, monitor worn time and BMI. (p for trend<p = 0.004).

Data from the Roots study suggested a negative association, with higher birth weight being associated with reduced sedentary time when adjusted for age, sex, SES, monitor worn time and BMI (β = −16.4 95%CI: −27.5, −5.3, p = 0.004). When displayed graphically, using quintiles of birth weight, the association seemed to be driven by the bottom two quintiles of birth weight (Figure 5).

**Figure 6:**
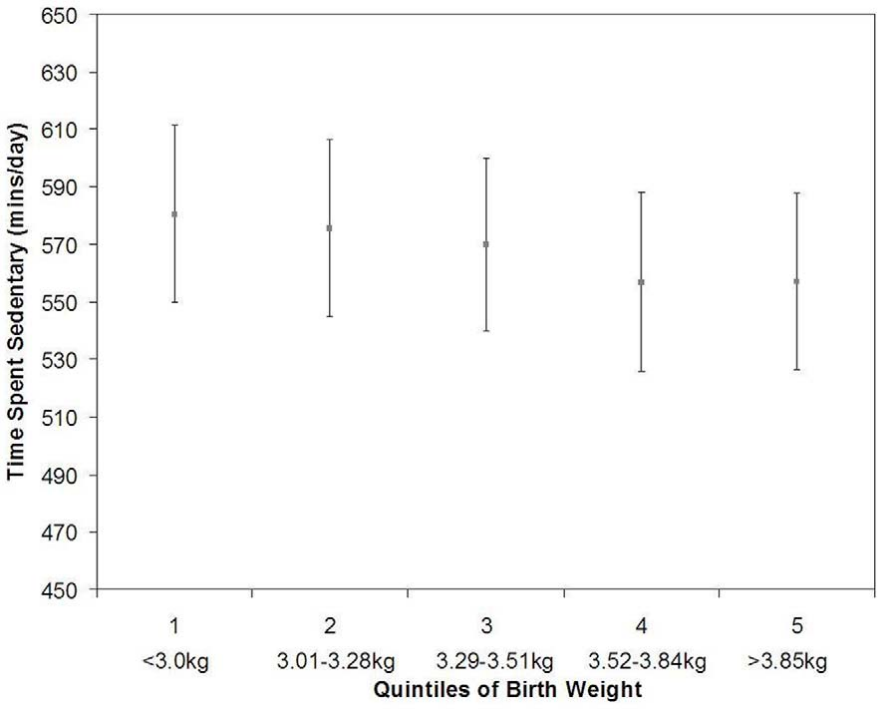
Association between quintiles of birth weight and sedentary time (mins/day) in the Pelotas Birth Cohort (n = 472). Means and 95% confidence intervals are adjusted for age, sex, SES, monitor worn time and BMI. (p for trend p = 0.004).

However, the data from the Pelotas study showed a positive association, with higher birth weight associated with greater sedentary time when adjusted for age, sex and monitor worn time (β = 17.0, 95%CI: 5.8, 28.2, p = 0.0031) (Table 3). This association was only slightly attenuated following additional adjustment for SES and BMI (β = 16.3, 95%CI: 5.2, 27.5 p = 0.004) (Table 3). It appears this association was largely driven by the lowest quintile of birth (Figure 6). However, further adjustment for gestational age attenuated the observed association (β = 12.2, 95%CI: −0.5, 25.0 p = 0.06) (Table 3).

## Discussion

While lower birth weight has previously been associated with reduced physical performance [11], aerobic fitness [12], [17], [32] and lower levels of self reported leisure time activity [14], our combined analysis did not find evidence that low birth weight predicts objectively measured habitual physical activity and sedentary time in healthy young people with a birth weight above 1.5 kg.

This is consistent with the results from a previous study in children and adolescents [21], which suggest that across the normal range of birth weights physical activity may be more influenced by environmental and behavioral factors. However, our observations may not be generalisable to very low birth weight infants since we excluded very low birth weight infants (<1.5 kg) from the present analyses. It is therefore still possible that very low birth weight or premature infants may have lower physical activity levels in later life [14], [18], [19].

Some of the individual studies within this combined analysis did however detect significant associations between birth weight and physical activity or sedentary time, which warrants further investigation. The data from the Roots study suggested an association between higher birth weight and lower sedentary time, with a 1 kg increase in birth weight equating to 16 mins less sedentary time during waking hours. This association appeared to be largely driven by those in the lower quintiles of birth weight. We are not aware of any other previous studies suggesting an association between lower birth weight and higher sedentary time, although self reported leisure time activity may be reduced in those at the lower end of the birth weight spectrum [14].

While in contrast, the results from the Pelotas study suggested that higher birth weight was associated with reduced total physical activity and increased sedentary time with approximately 16 minutes increased sedentary time during waking hours per 1 kg increase in birth weight. These findings in Pelotas are in the opposite direction to the hypothesized association, such as the lower self reported leisure time activity in those born with very low birth weight [14], [18].

As the Pelotas study collected data close to the time of birth we were able to further adjust the analysis for gestational age. The associations between birth weight and both sedentary time and total activity, were attenuated and no longer significant with additional adjustment for gestational age. These findings suggest that the associations in Pelotas may be mediated via gestational age or simply that those born at the lower end of the birth weight spectrum are most likely to also be born with a shorter gestation, as birth weight and gestational age were correlated (r = 0.4, p<0.001).

Since higher birth weight is associated with both increased BMI [33] and increased fat mass [34] in children, we hypothesized that the reduced physical activity associated with higher birth weight may be due to increased adiposity. We repeated the models for the Pelotas data adjusting for percentage body fat (measured by deuterium dilution) but the findings were largely unchanged (data not shown), suggesting that this association is not mediated via differences in adiposity composition.

It was also possible that these findings may be driven by differences in the collection and processing of the physical activity data. The mean and SD for total activity, time spent in MVPA and sedentary time for the Roots and Pelotas studies were very comparable. However both the Pelotas study and the Roots study had 24 hour monitor wear protocols. While we attempted to standardise the studies by excluding overnight data (between the hours of 22:59 hours and 07:00 hours) this may have influenced the findings, particularly if there are country specific differences in waking and sleeping hours. For example there is evidence that teenagers in western societies displace sleep with increased sedentary activities, such as computer use [35], whereas children in a developing country such as Brazil may have differing patterns of time use. To investigate this further we reanalyzed the Pelotas dataset, without excluding the overnight data. While including overnight data meant the absolute values for sedentary time were substantially increased, because of the longer wear time, the overall findings were in the same direction and of similar magnitude (data not shown). It is therefore unlikely that the observation of a positive association between birth weight and sedentary time in the Pelotas study is due to the wear protocol or data processing.

It is possible that the disparate findings from the Pelotas study and the Roots study may represent genuine differences between these populations, as Pelotas is from a developing country, whereas the participants in the Roots study are from a generally affluent region of the UK (Cambridgeshire), so may experience very different in utero environments as well as differences in physical activity patterns. However it should be recognised that, although statistically significant, the magnitude of the associations between birth weight and physical activity and sedentary time are very small and may not be clinically relevant. For example, the data from the Pelotas study suggested that an increase in birth weight of 1 kg was associated with 31 counts per minute less total physical activity. This corresponds to less than one fifth of a SD unit. Similarly, 1 kg higher birth weight was associated with about 16 minutes (2.9%) more sedentary time per day in the ROOTs study, so while the findings are statistically significant they may not be relevant in practical terms.

However there are some limitations to this combined analysis, which should be considered when interpreting the findings. For three of the four studies, birth weight was retrospectively reported. Studies suggest that maternally reported birth weight is particularly well recalled and correlates highly with measured birth weight [36]. However without adjustment for gestational age we cannot preclude that the observed associations are not confounded by gestational age. We attempted to minimise the influence of premature infants by excluding those born with very low birth weight (<1.5 kg) since these infants are most likely to be premature. Finally, it should also be noted that while the analysis were adjusted for SES this variable was parentally self-reported in all the studies and there were differences in how SES was measured and classified between the studies, so some degree of residual confounding may persist. However this analysis is considerably strengthened by including population based cohorts from very differing countries, as well as including objective measures of physical activity.

This present analysis was limited to those born in the low to normal weight spectrum of birth weights. The recent meta-analysis of self-reported leisure time activity suggests that both low and high birth weight extremes are associated with lower leisure time physical activity [14]. It would be valuable to use objective monitoring to investigate whether individuals with more extreme growth restriction, such as those born with very or extremely low birth weight actually are less active than their normal-weight peers. It would also be particularly useful to use studies with information on gestational age, so it would be possible to differentiate the influence of growth restriction, such as small for gestation age, from those born prematurely especially given the increased survival of both low birth weight and premature infants and their increased risk of metabolic disease [18]. Furthermore, given the findings of Andersen *et al* [14], which suggest that there may also be an association between very high birth weight and lower self-reported leisure time physical activity, with the current increasing prevalence of childhood obesity further studies are needed to examine whether very high birth weight, such as in macrosomic infants, is associated with lower levels of later physical activity and higher levels of sedentary time. There may also be other early life variables which could also act as biological determinants of physical activity or sedentary behavior. For example both rapid infant weight gain and slower infant motor development have been associated with reduced muscle strength and aerobic fitness in adulthood [11], which could in theory extend to acting on habitual activity levels. Prospective studies with intermediate measures would be particularly beneficial to elucidate potential pathways involved, such the influence of growth and development and the role of physical capacity and fitness. For example Rogers *et al* [17] found similar levels of enjoyment of sports and physical activity between those born with extremely low birth weight and their normal weight peers, however those born with very low birth weight did have poorer motor co-ordination. Finally it is possible that behavioral aspects may influence the birth weight and physical activity relationship, such as more protective parenting in those born with very low birth weight or where infants are born premature.

Overall this combined analysis suggests that birth weight is not an important biological determinant of habitual physical activity or sedentary behaviour in children and adolescents. This reassuring finding suggests that although lower birth weight may reduce physical capacity in later life, this does not extend to reducing levels of habitual physical activity.
